# Supplementary material for: Critical role of water structure around interlayer ions for ion storage in layered double hydroxides
Source: Nat Commun. 2022 Oct 28;13:6448. doi: 10.1038/s41467-022-34124-9 (PMC9616869; doi:10.1038/s41467-022-34124-9)
Supplement: Supplementary file 1 — Supplementary Information [file 41467_2022_34124_MOESM1_ESM.docx]

**Supplementary Information**

**Critical role of water structure around interlayer ions for ion storage in layered double hydroxides**

Tomohito Sudare^1^*, Takuro Yamaguchi^2^, Mizuki Ueda^2^, Hiromasa Shiiba^1^, Hideki Tanaka^1^, Mongkol Tipplook^1^, Fumitaka Hayashi^2^ & Katsuya Teshima^1,2^*

^1^ Research Initiative for Supra-Materials (RISM), Shinshu University, 4-17-1 Wakasato, Nagano 380-8553, Japan

^2^ Department of Materials Chemistry, Faculty of Engineering, Shinshu University, 4-17-1 Wakasato, Nagano 380-8553, Japan

**Contents:**

**Page 2 Supplementary Equations 1–3**

**Page 3–17 Supplementary Figures 1–18**

**Page 18 Supplementary Table 1**

**Page 19 Supplementary References 1–6**

**Typical isotherm models:**

The ion-exchange adsorption isotherms were analysed using the following three models^1,2^:

Langmuir model

$\boldsymbol{q}\mathbf{=}\frac{\boldsymbol{q}_{\boldsymbol{m}}\boldsymbol{K}_{\boldsymbol{L}}\boldsymbol{C}_{\boldsymbol{e}}}{\mathbf{1+}\boldsymbol{K}_{\boldsymbol{L}}\boldsymbol{C}_{\boldsymbol{e}}}$ (1)

Freundlich model

$\boldsymbol{q}\mathbf{=}\boldsymbol{K}_{\boldsymbol{F}}\boldsymbol{C}_{\boldsymbol{e}}^{\mathbf{1/}\boldsymbol{d}}$ (2)

Langmuir−Freundlich (LF) model

$\boldsymbol{q}\mathbf{=}\frac{{\boldsymbol{q}_{\boldsymbol{m}}\mathbf{(}\boldsymbol{K}_{\boldsymbol{LF}}\boldsymbol{C}_{\boldsymbol{e}}\mathbf{)}}^{\mathbf{1/}\boldsymbol{n}}}{\mathbf{1+}{\mathbf{(}\boldsymbol{K}_{\boldsymbol{LF}}\boldsymbol{C}_{\boldsymbol{e}}\mathbf{)}}^{\mathbf{1/}\boldsymbol{n}}}$ (3)

where *q* (mmol mol^−1^) is the amount of the cations adsorbed at the equilibrium concentration, *q*_m_ is the maximum amount of adsorbed sorbent for the Langmuir and LF models, *K*_L_ and *K*_LF_ are the Langmuir and LF constants, respectively, *C*_e_ (mmol L^−1^) is the equilibrium concentration, *K*_F_ is the Freundlich constant, and *d* and *n* are constants.

**Figures:**


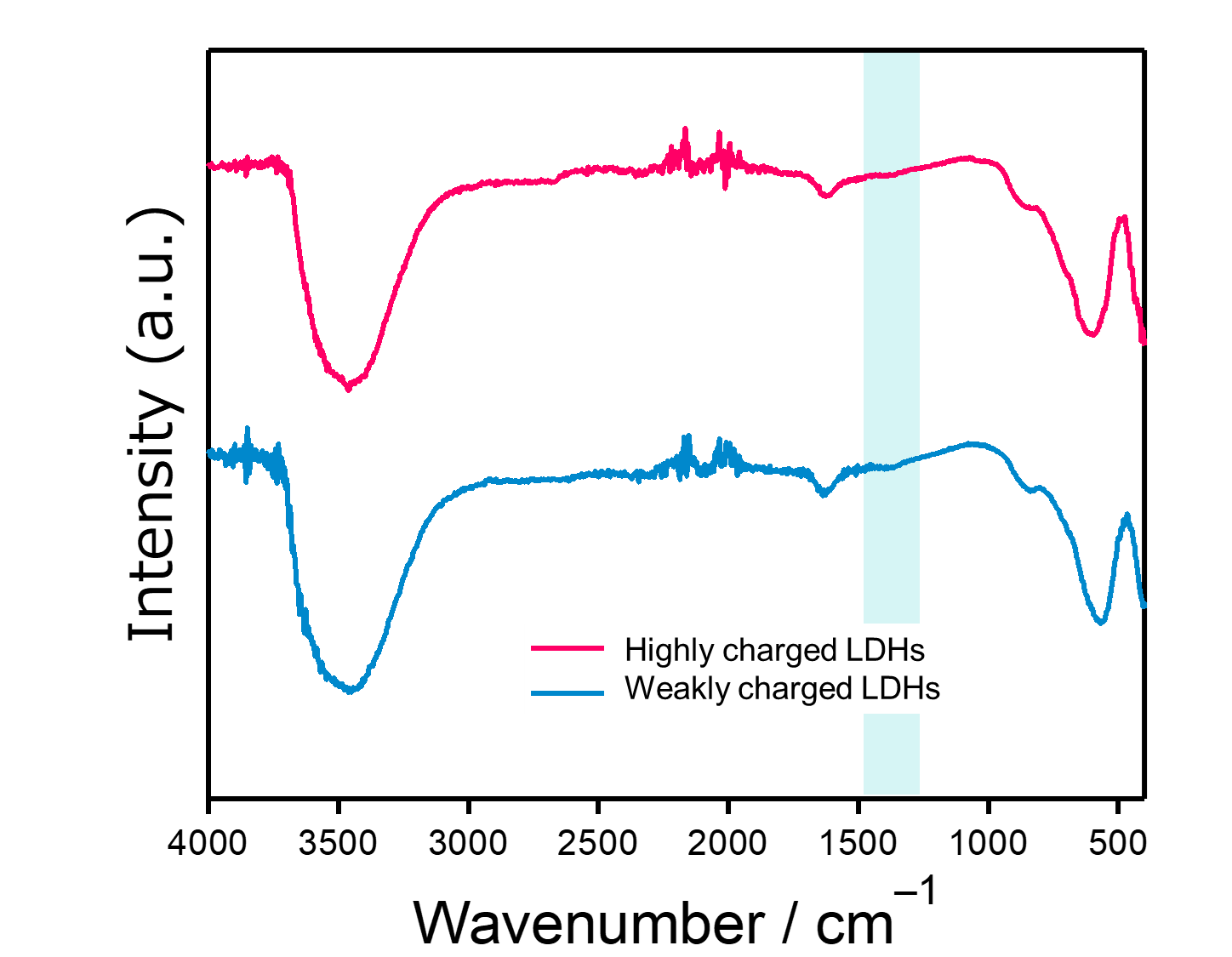


**Supplementary Fig. 1 |** FT-IR ATR spectral profiles recorded for the as-prepared highly and weakly charged LDHs. The successful removal of interlayer carbonate ions is confirmed in both samples by the absence of the asymmetric *ν*_3_ CO_3_ band (at 1460 cm^−1^)^3–6^.


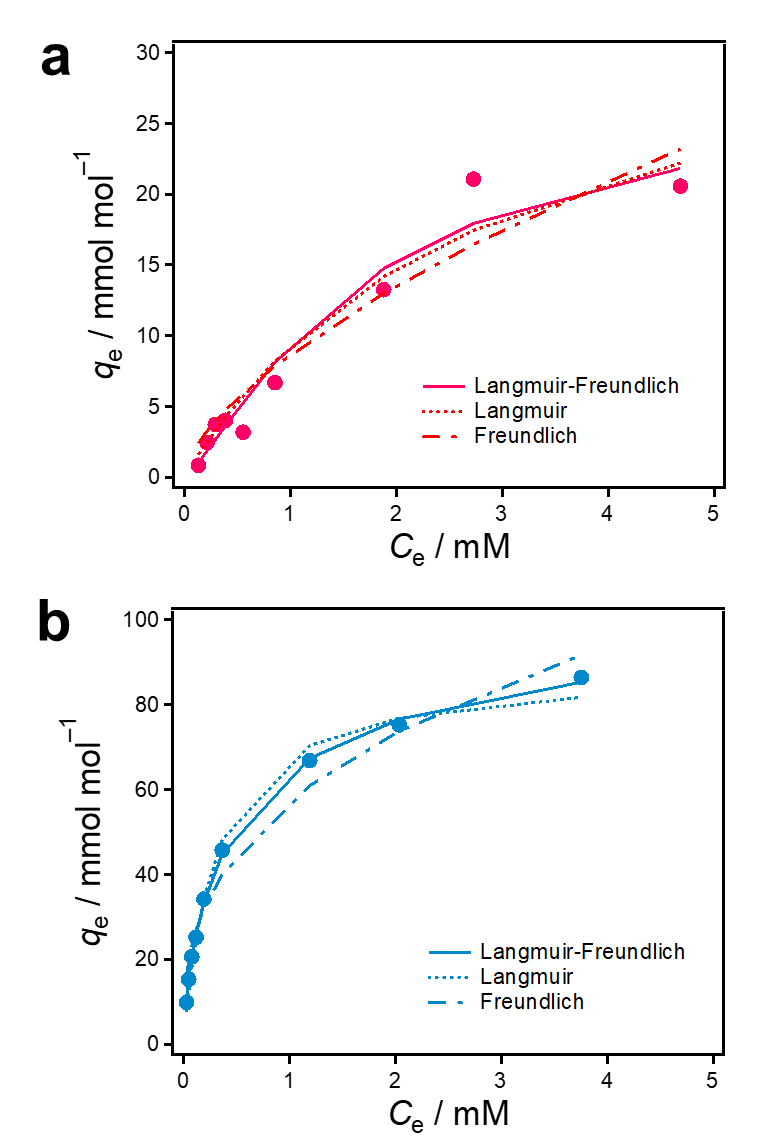


**Supplementary Fig. 2 |** Curve fits corresponding to the anion-exchange adsorption isotherms in the dilute region (Fig. 2c), obtained using the Langmuir, Langmuir–Freundlich (LF), and Freundlich models for the as-prepared highly (**a**) and weakly (**b**) charged LDHs.


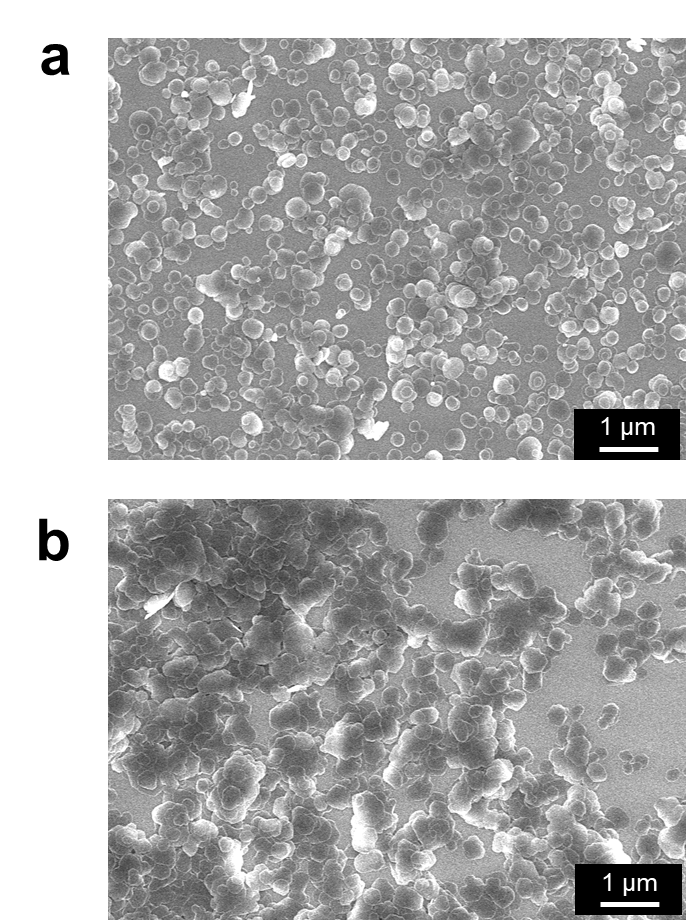


**Supplementary Fig. 3 |** FE-SEM images of thin films of highly (**a**) and weakly charged LDHs (**b**) deposited on SiO_2_-coated Au electrode (QCM-D sensor).


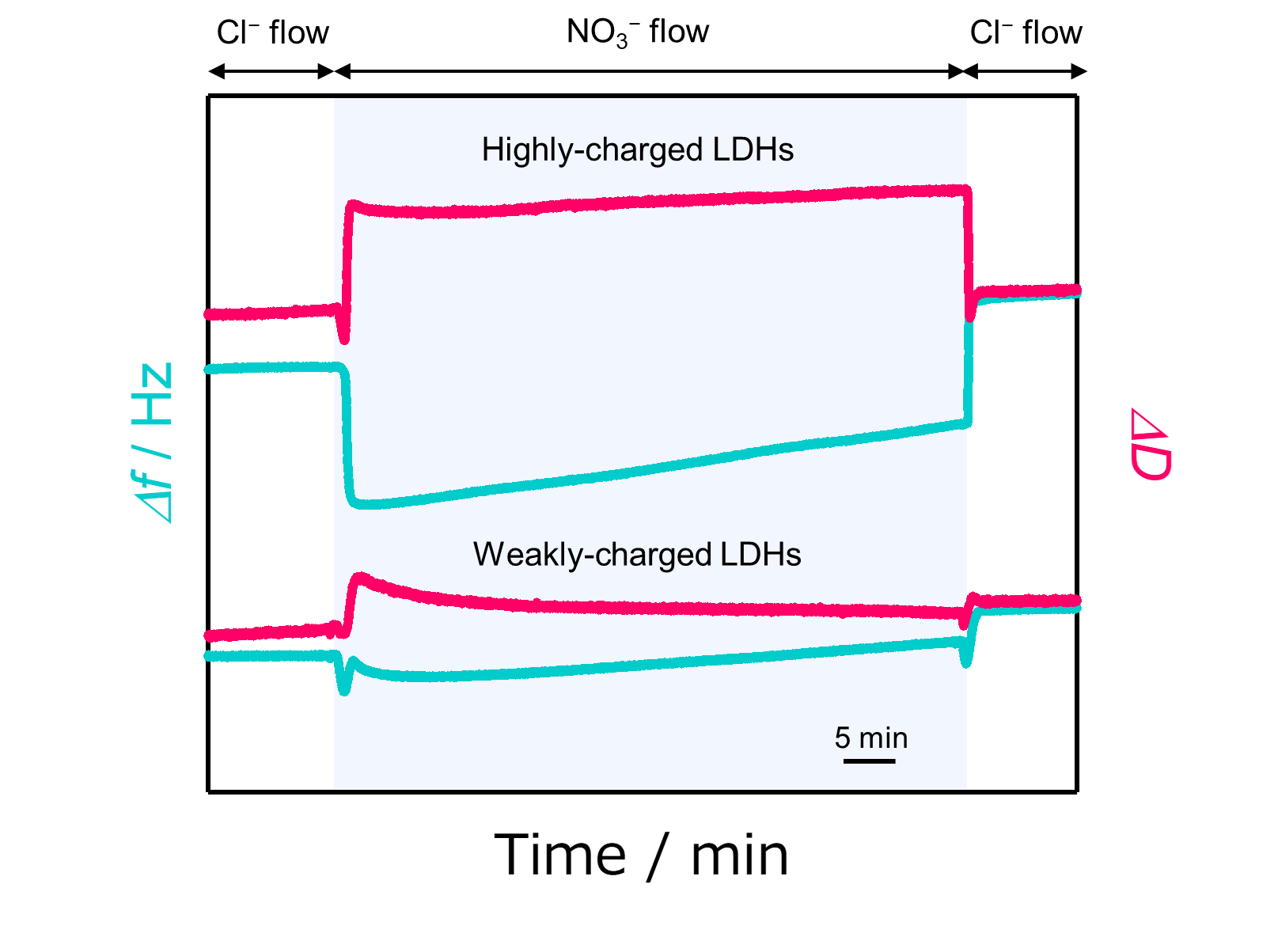


**
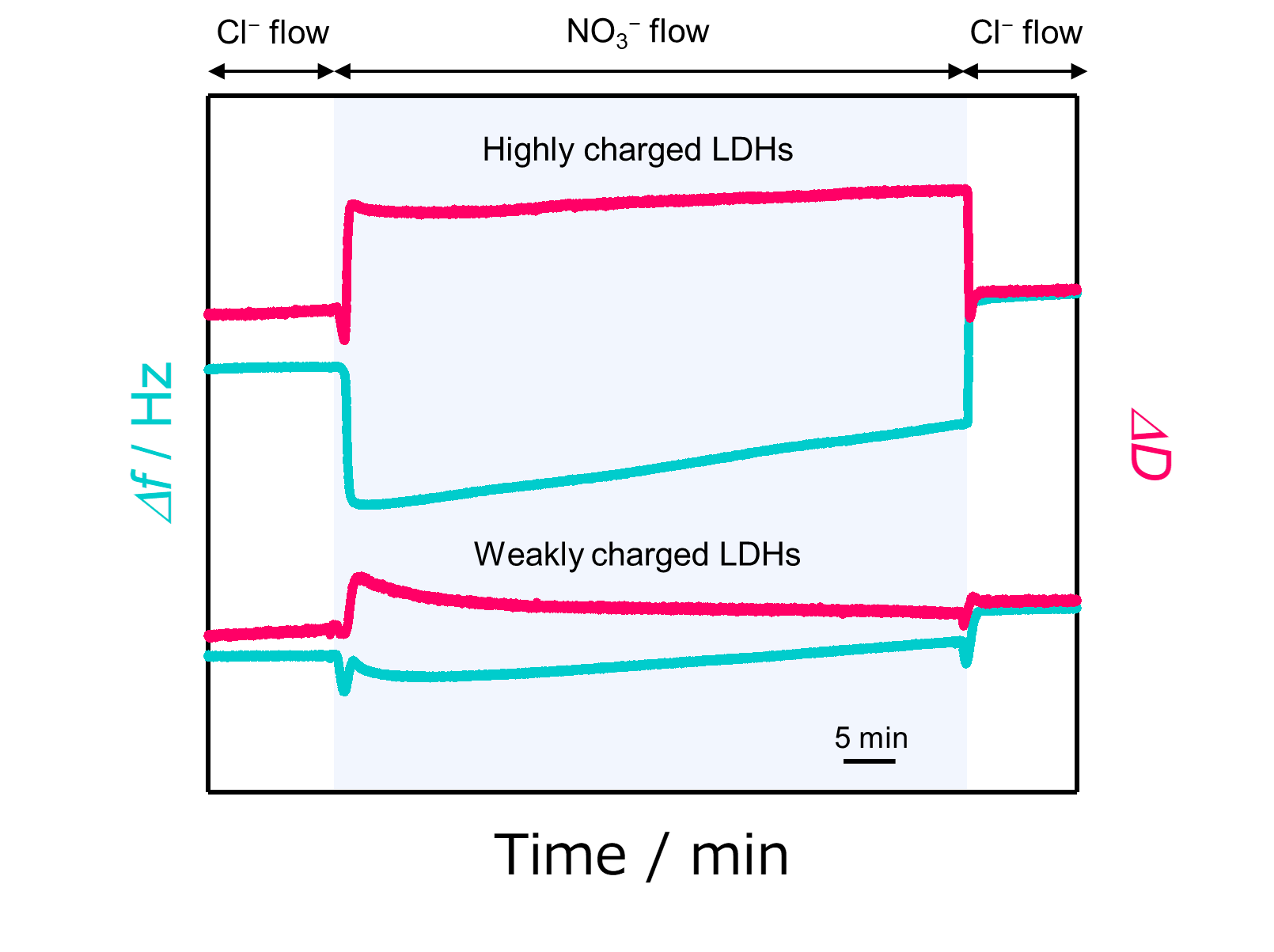
**

**Supplementary Fig. 4 |** Expanded QCM-D profiles indicated in Fig. 3b. The scale of the Y-axis is the same as in Fig. 3.


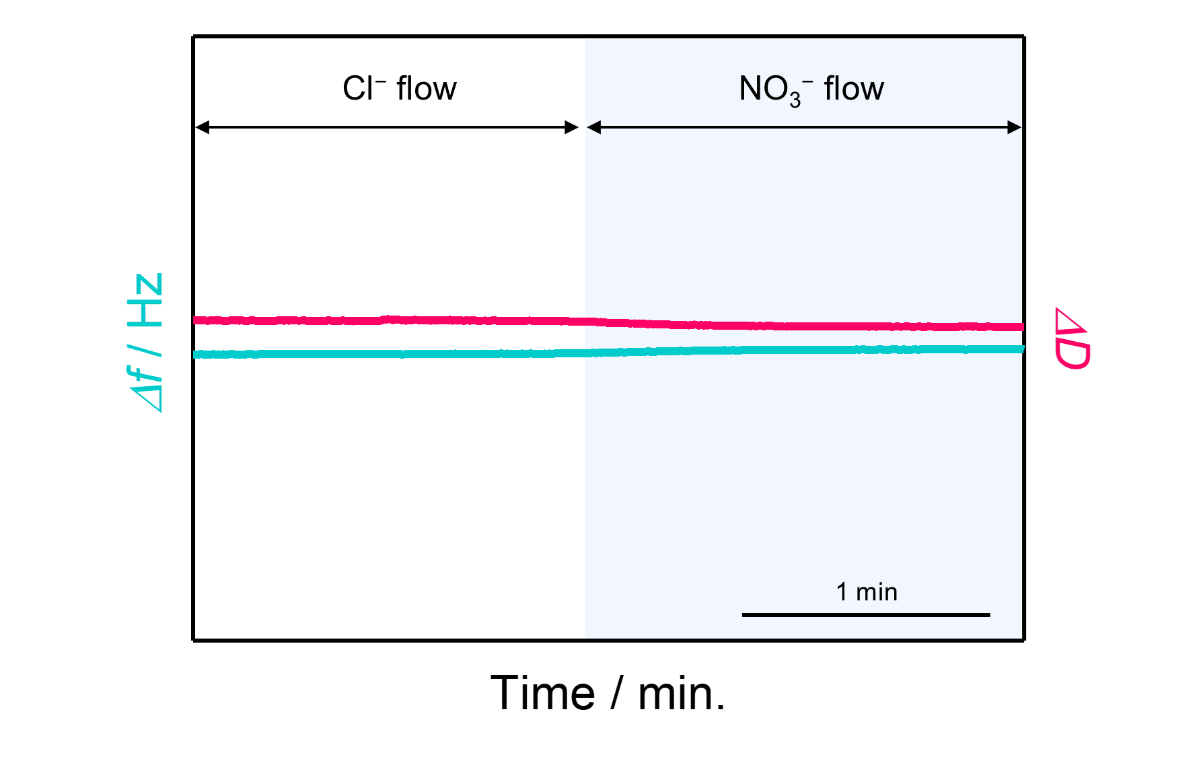


**Supplementary Fig. 5 |** Real-time monitoring of the changes in the frequency (*Δf*) and dissipation (*ΔD*) by QCM-D measurement upon changing the test solution from aqueous NaCl solution to NaNO_3_ solution with respect to SiO_2_-coated Au electrode (QCM-D sensor) without deposited LDH thin film. The flow rate is set to 300 μL min^–1^. The scale of the X- and Y-axes is the same as in Fig. 3.


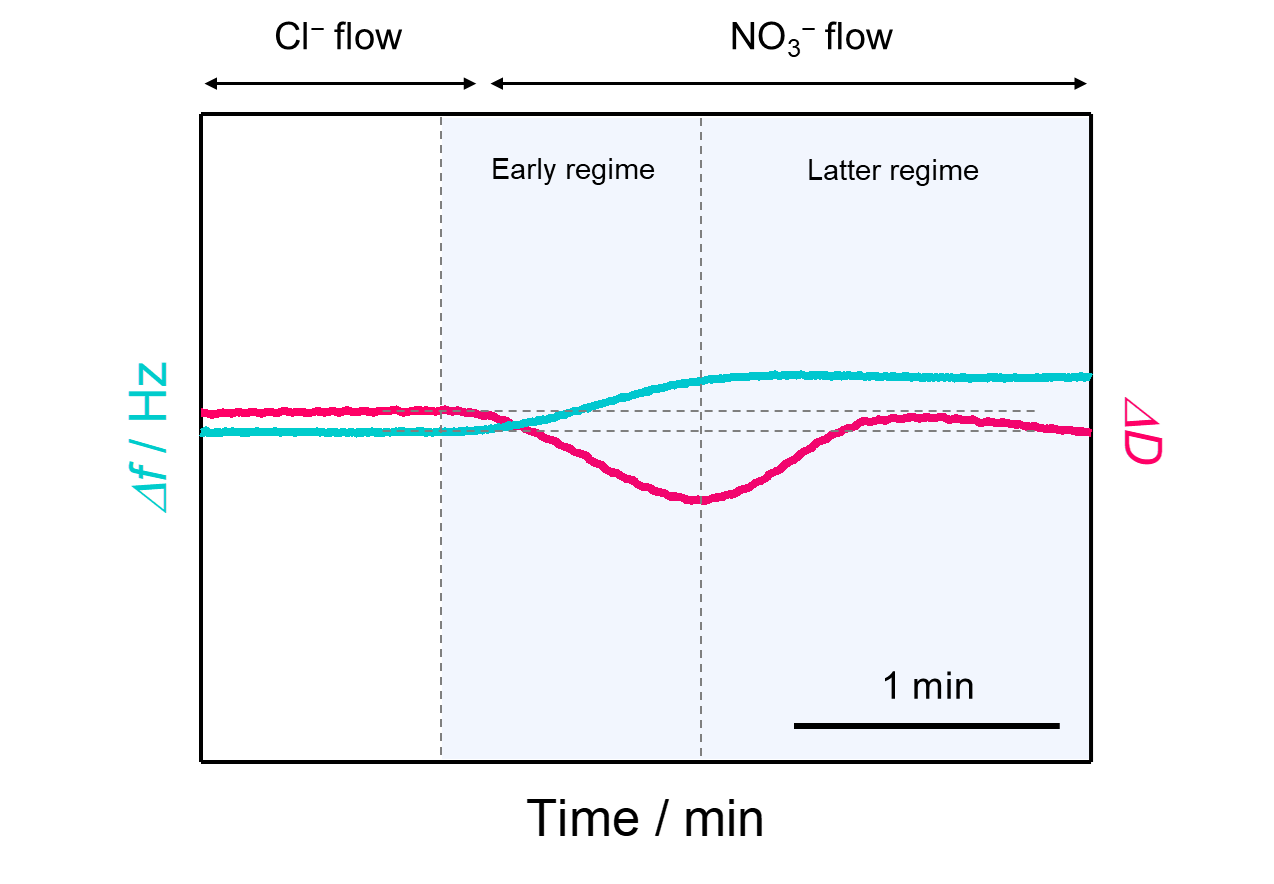


**Supplementary Fig. 6 |** Real-time monitoring of the changes in the frequency (*Δf*) and dissipation (*ΔD*) by QCM-D measurement upon changing the test solution from aqueous NaCl solution to NaNO_3_ solution with respect to the as-prepared highly charged LDH thin film. The flow rate is set to 200 μL min^–1^. The scale of the X- and Y-axes is the same as in Fig. 3.


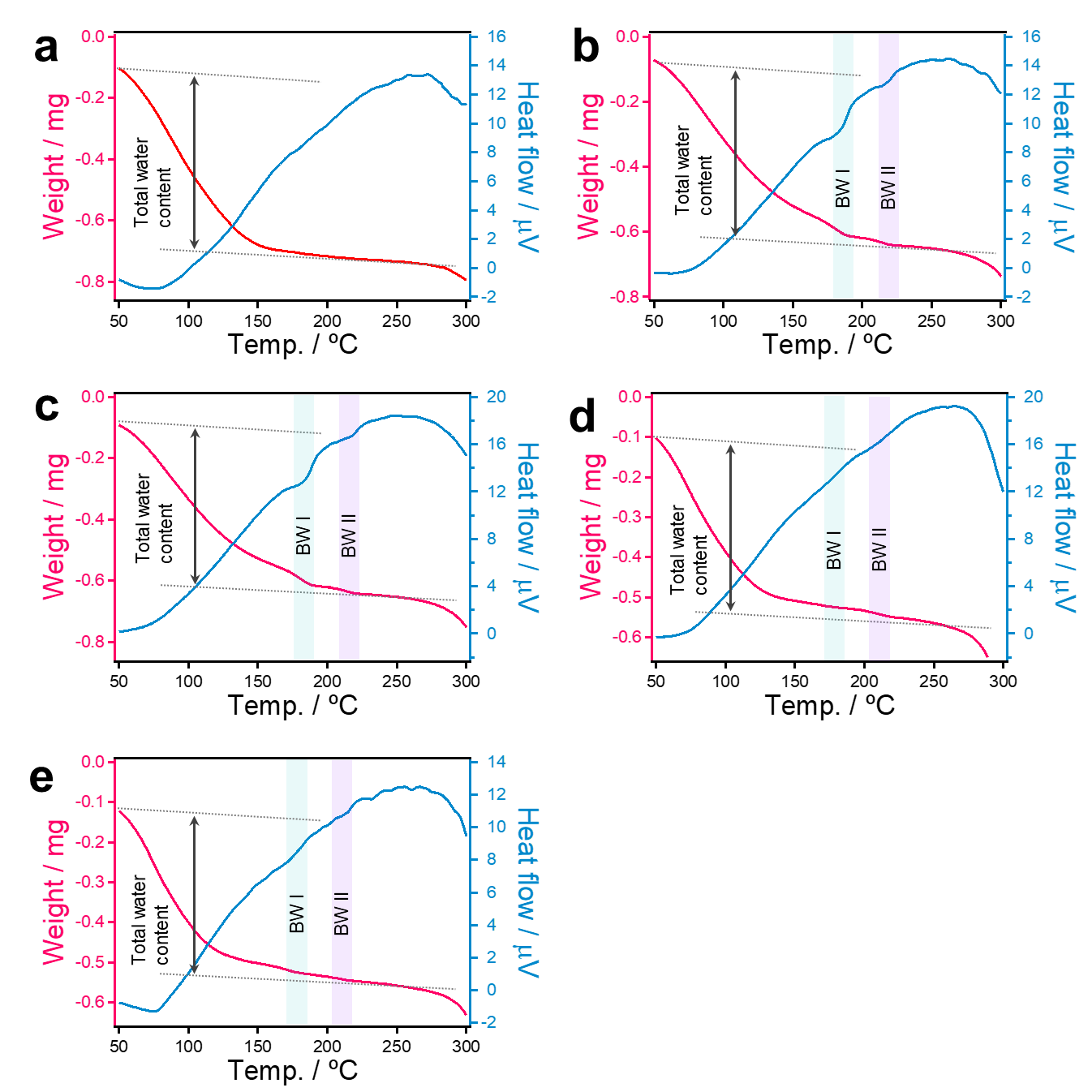


**Supplementary Fig. 7 |** Thermogravimetric-differential thermal analysis (TG-DTA) profiles of the highly charged LDHs, I (**a**), II (**b**), III (**c**), IV (**d**), and V (**e**).


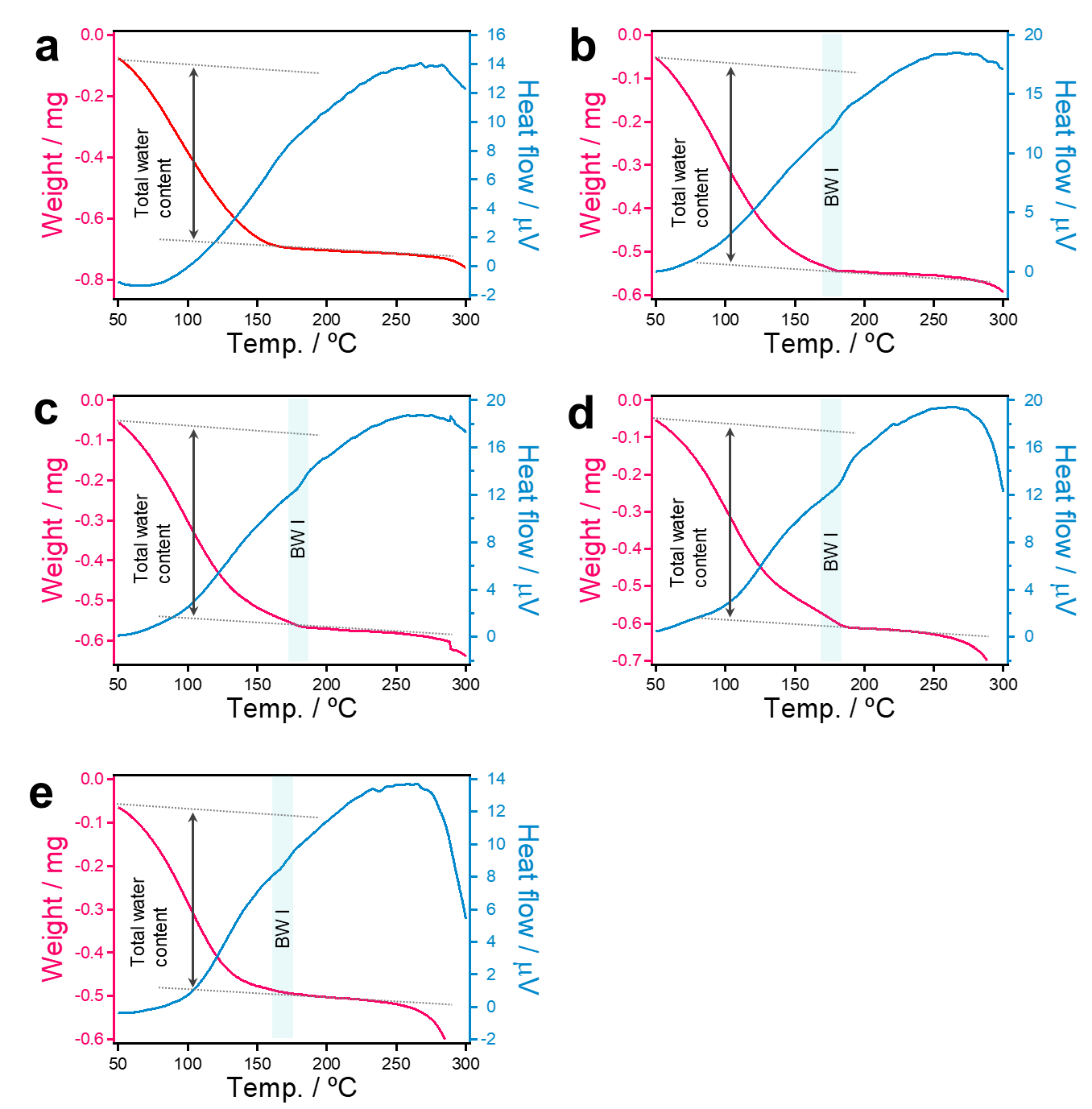


**Supplementary Fig. 8 |** TG-DTA profiles of the weakly charged LDHs, I (**a**), II (**b**), III (**c**), IV (**d**), and V (**e**).


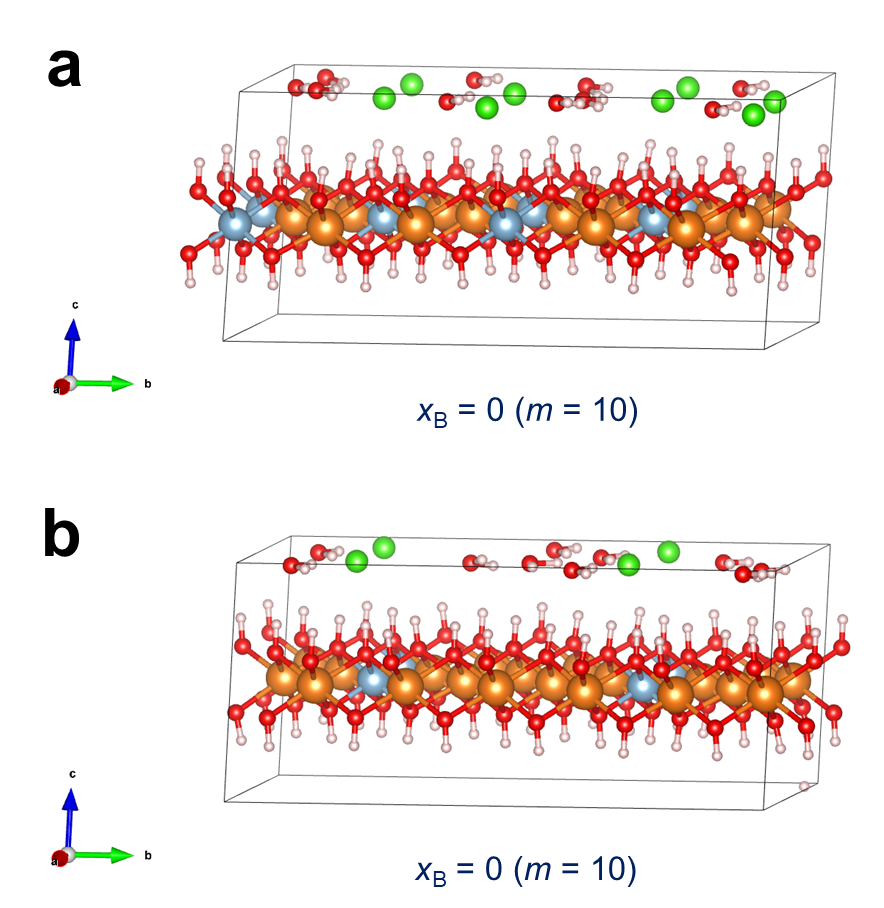


**Supplementary Fig. 9 |** Examples of the original structures of [Mg_0.66_Al_0.33_(OH)_2_](Cl)_0.33_·0.42H_2_O (**a**) and [Mg_0.833_Al_0.166_(OH)_2_](Cl)_0.166_·0.42H_2_O (**b**) used for DFT calculations.


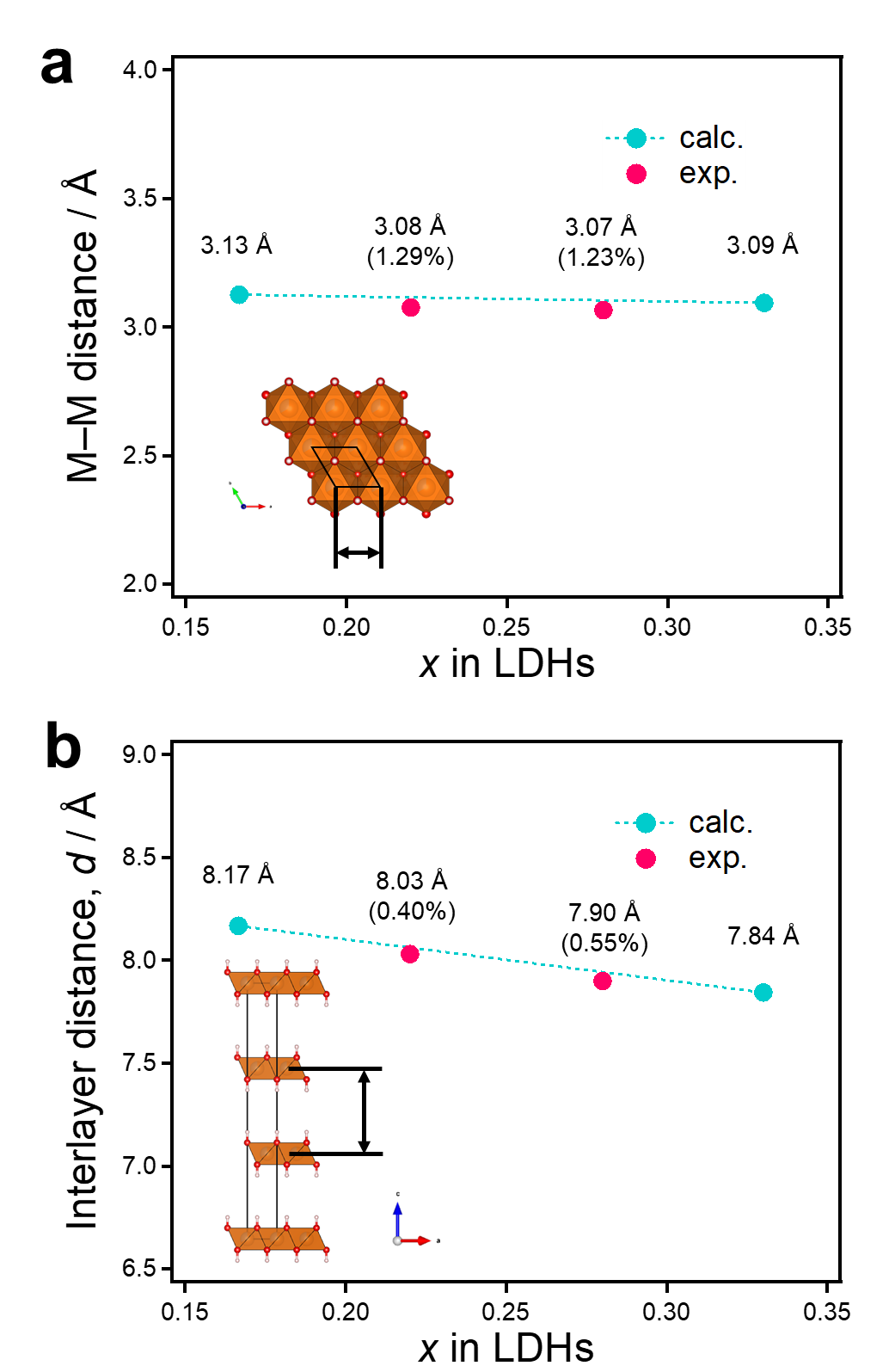


**Supplementary Fig. 10 |** Correspondence of the lattice parameters (the nearest metal cation distance in the host-layer (**a**) and interlayer distance between the optimized Mg/Al LDH structure (**b**)) obtained by DFT calculations (calc.) and the as-prepared (exp.) Mg/Al LDHs. The relative error is indicated in parentheses.


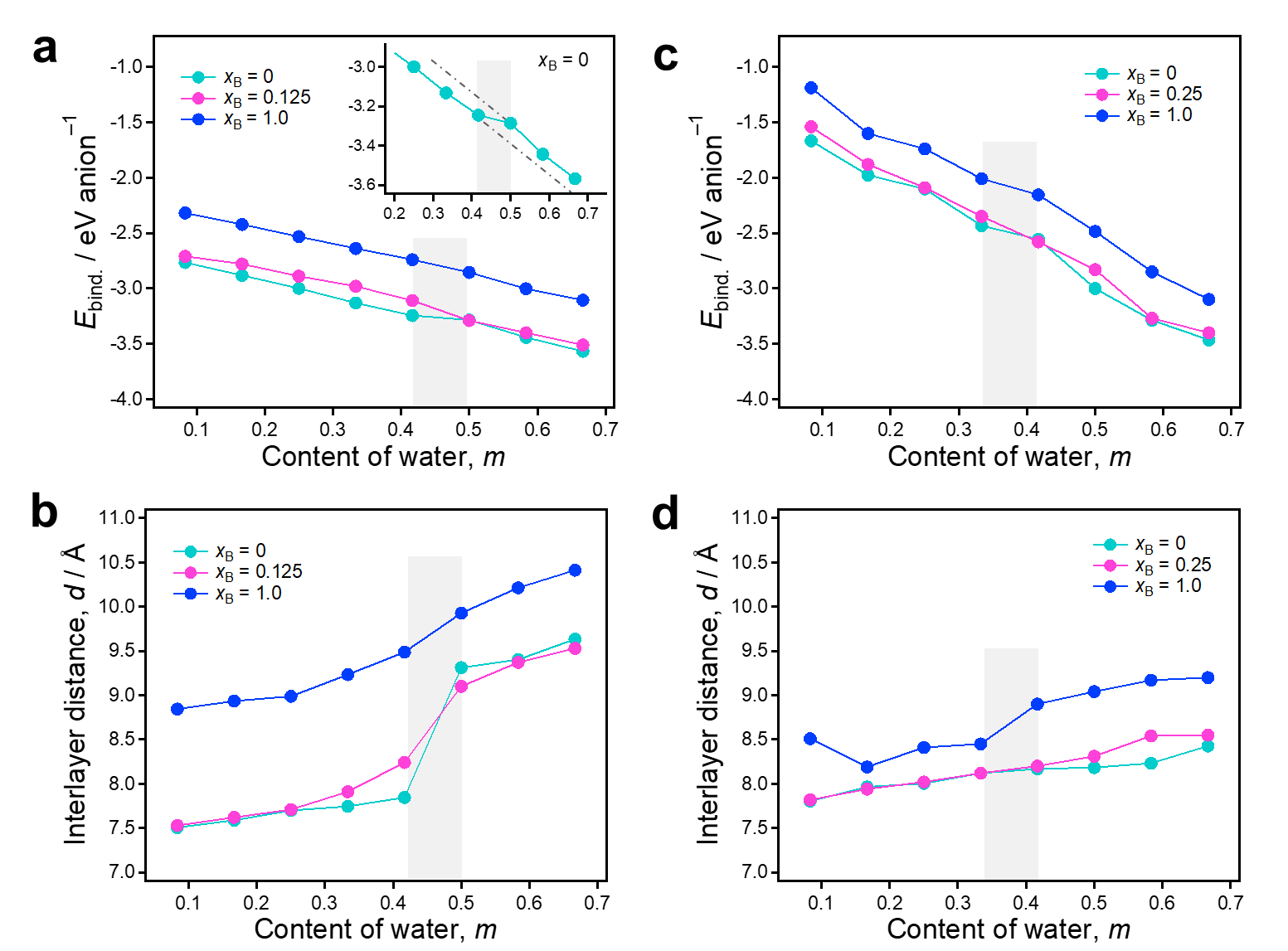


**Supplementary Fig. 11 |** The interlayer binding energy and interlayer distance as a function of water content (*m*) for the optimized Mg/Al LDH structure (**a**, **b**) with highly charged [Mg_0.66_Al_0.33_(OH)_2_] layers and (**c**, **d**) with weakly charged [Mg_0.83_Al_0.166_(OH)_2_] layers.


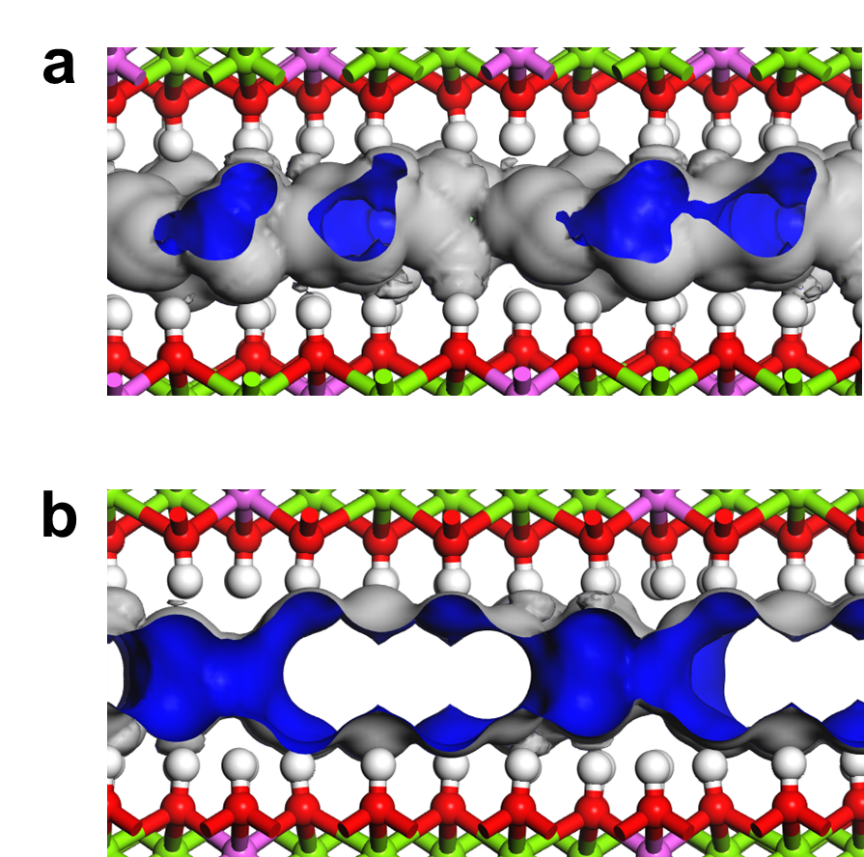


**Supplementary Fig. 12 |** Illustration of the Connolly surface of the structures presented in Supplementary Figs. 9a and 9b calculated using a Connolly radius of 1.0 Å, where the water molecules have been removed.


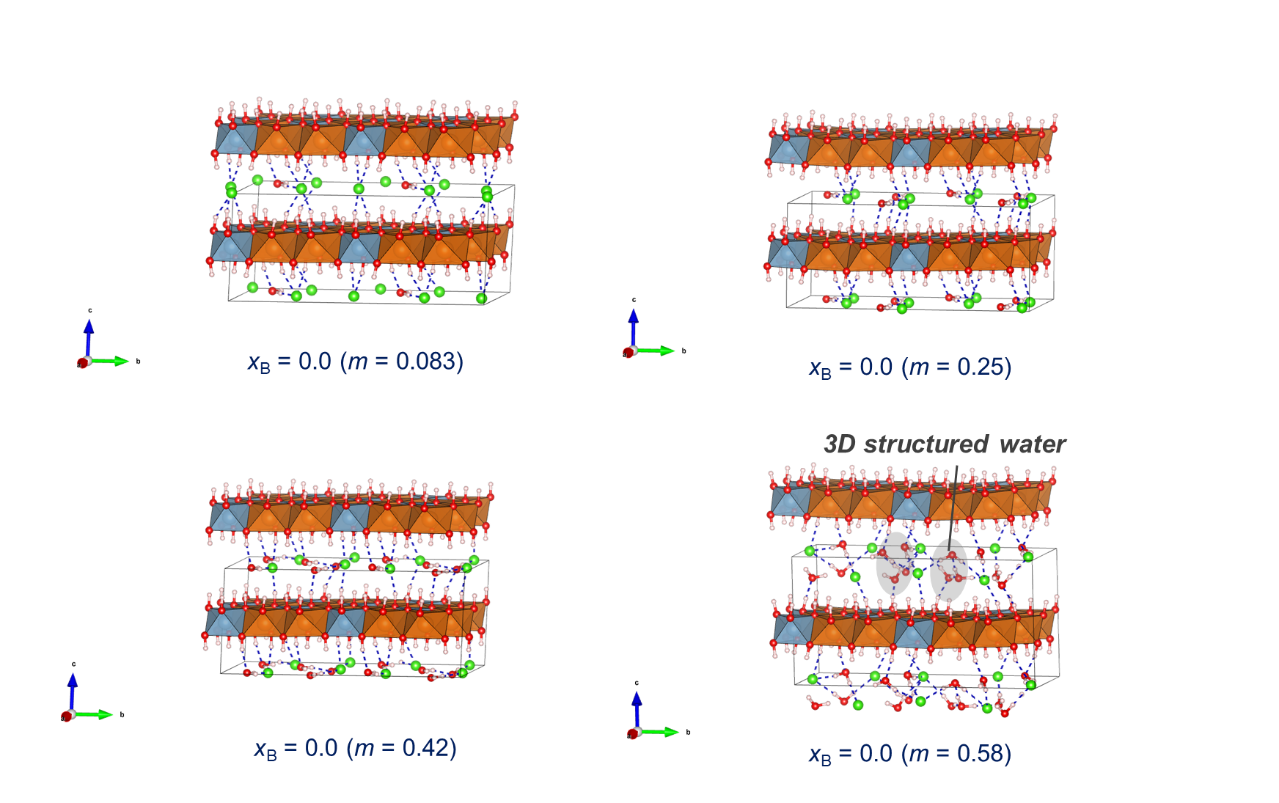


**Supplementary Fig. 13 |** The optimized Mg/Al LDH structures with the highly charged [Mg_0.66_Al_0.33_(OH)_2_] layers for *x*_B_ = 0.0.


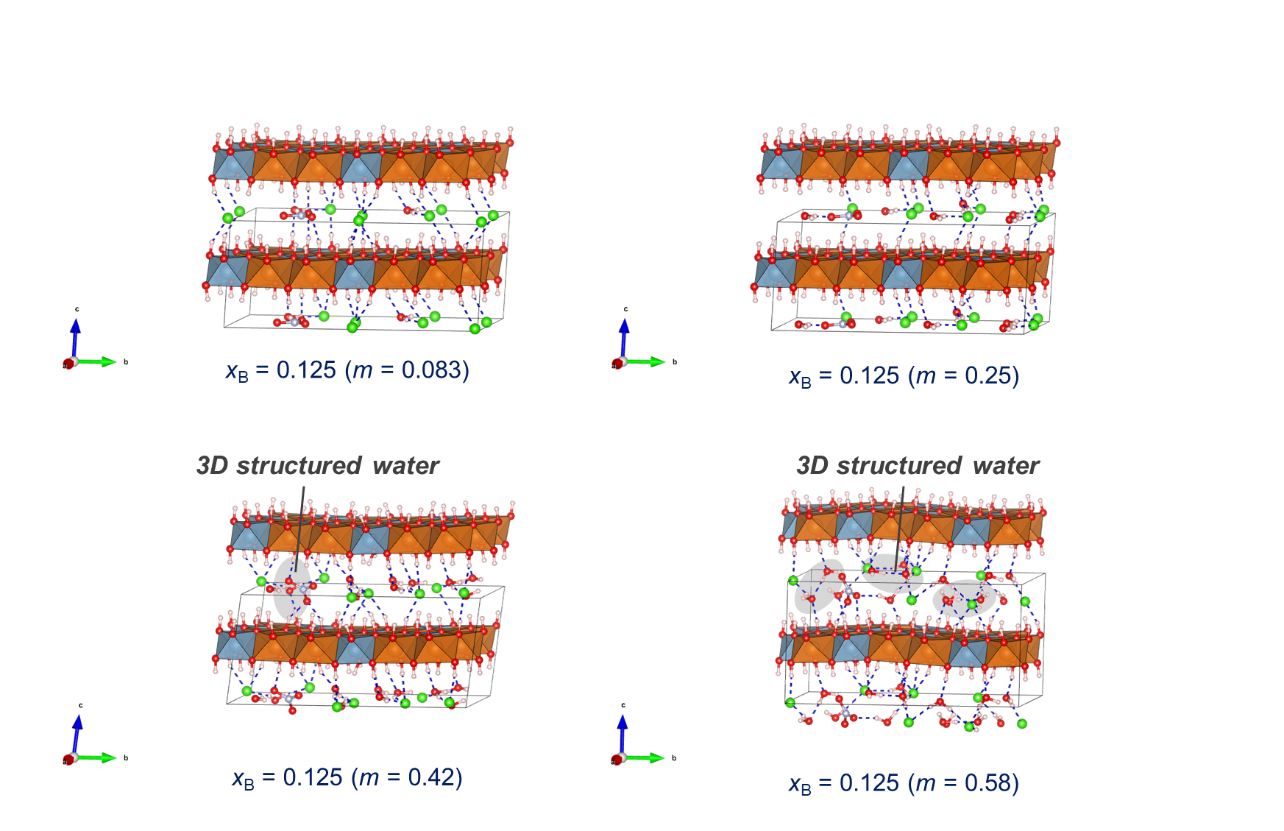


**Supplementary Fig. 14 |** The optimized Mg/Al LDH structures with the highly charged [Mg_0.66_Al_0.33_(OH)_2_] layers for *x*_B_ = 0.125.


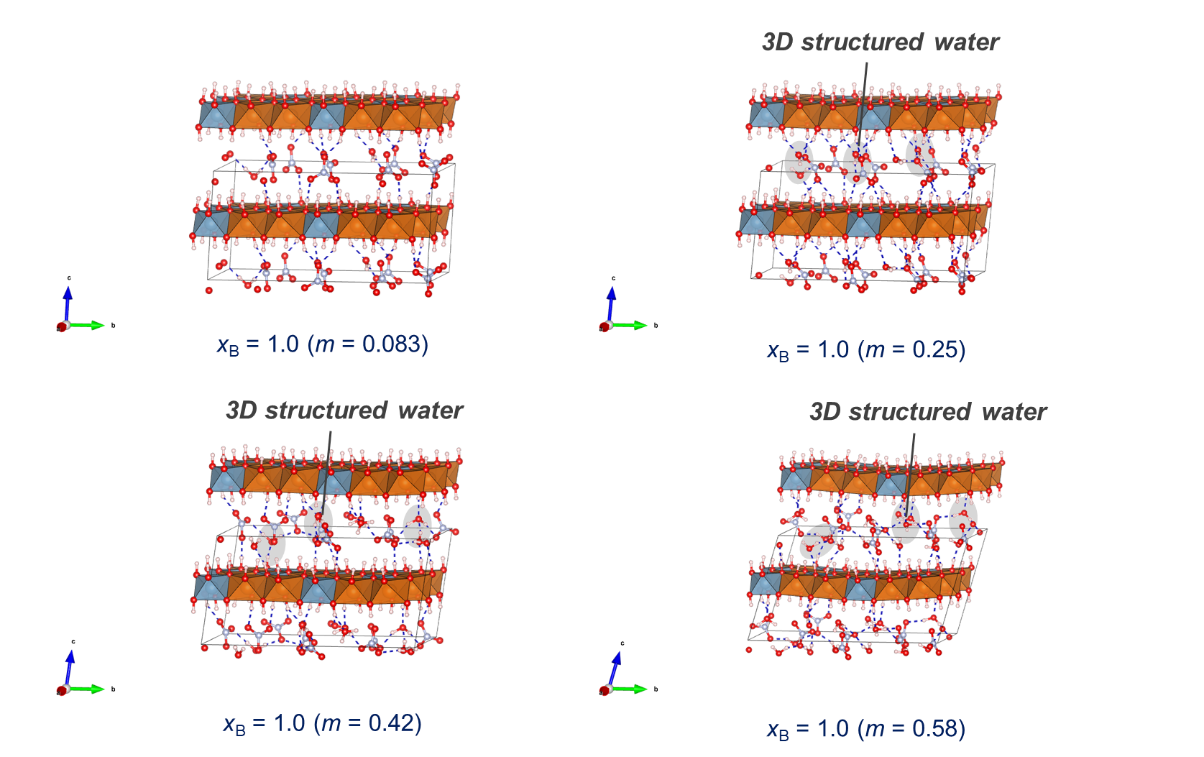


**Supplementary Fig. 15 |** The optimized Mg/Al LDH structures with the highly charged [Mg_0.66_Al_0.33_(OH)_2_] layers for *x*_B_ = 1.0.


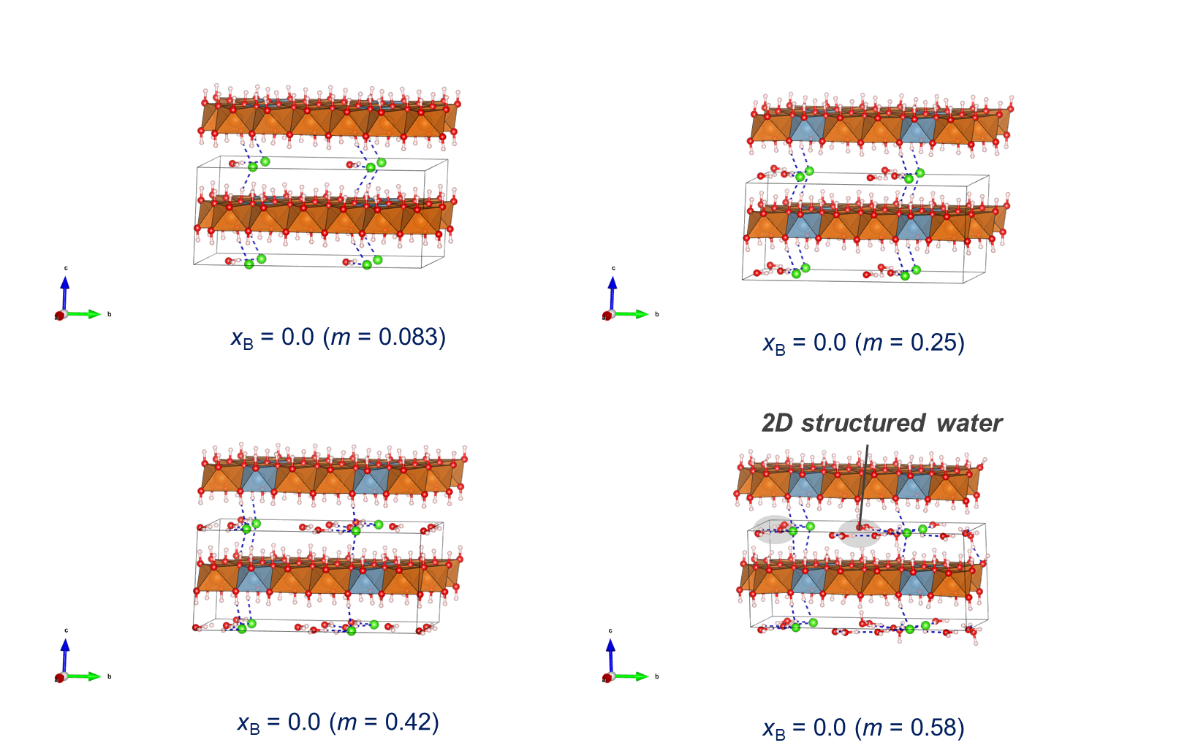


**Supplementary Fig. 16 |** The optimized Mg/Al LDH structures with the weakly charged [Mg_0.833_Al_0.166_(OH)_2_] layers for *x*_B_ = 0.0.


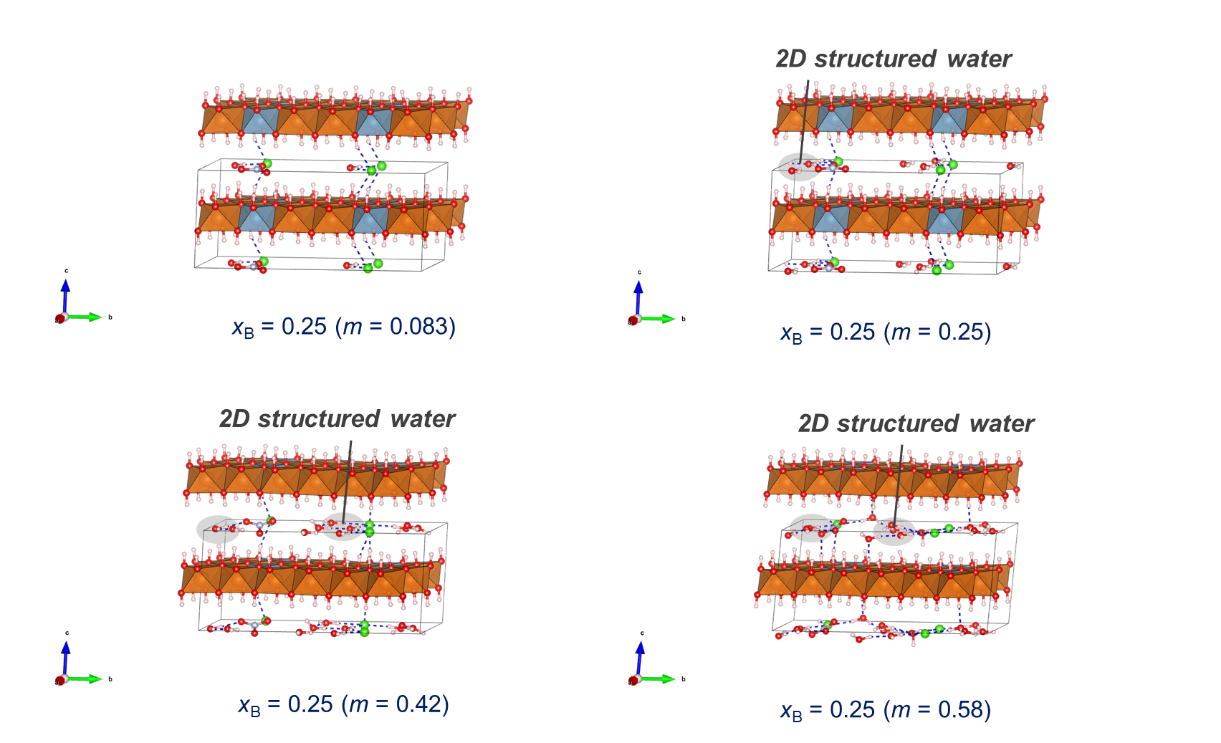


**Supplementary Fig. 17 |** The optimized Mg/Al LDH structures with the weakly charged [Mg_0.833_Al_0.166_(OH)_2_] layers for *x*_B_ = 0.25.


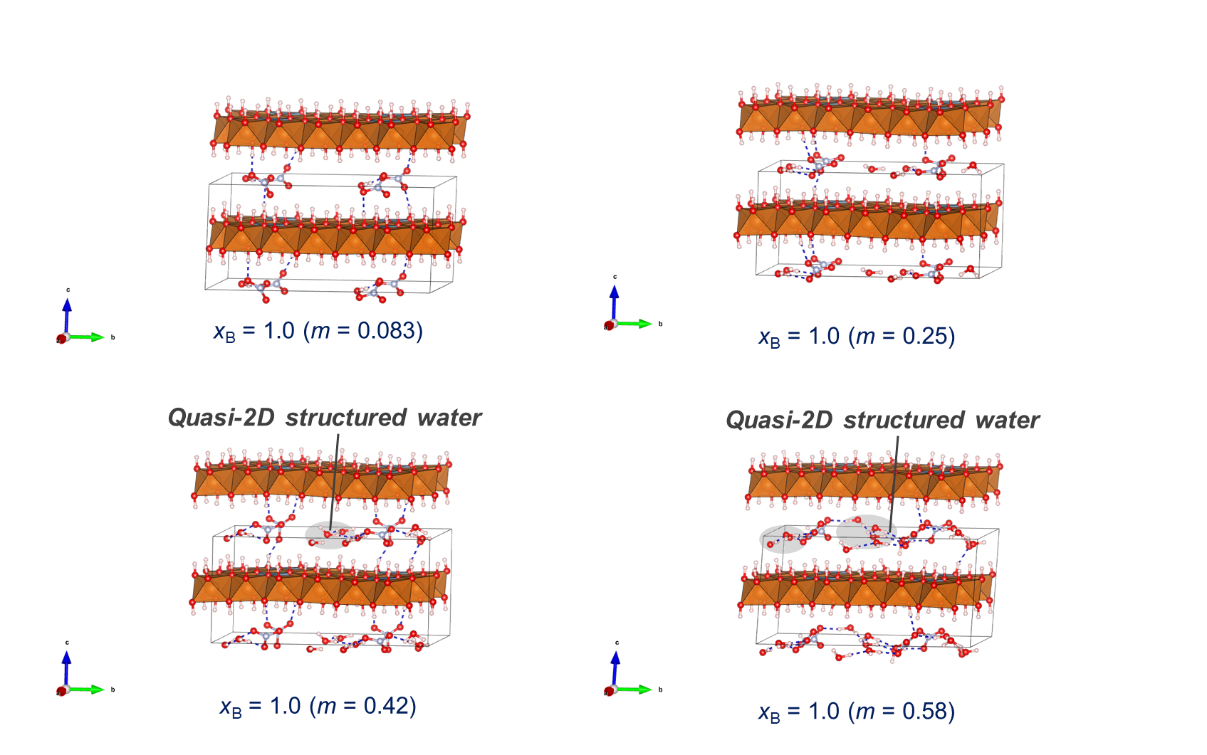


**Supplementary Fig. 18 |** The optimized Mg/Al LDH structures with the weakly charged [Mg_0.833_Al_0.166_(OH)_2_] layers for *x*_B_ = 1.0.

**Tables:**

**Supplementary Table 1 |** Parameters obtained from the isotherm fits of the as-prepared Mg/Al LDHs. The fitting results for the dilute region are shown in Supplementary Fig. 1.

|  | Langmuir | | |  | Langmuir–Freundlich | | | |  | Freundlich | | |
| --- | --- | --- | --- | --- | --- | --- | --- | --- | --- | --- | --- | --- |
|  | *q*_m_ | *K*_L_ | *R^2^* |  | *n* | *q*_m_ | *K*_LF_ | *R^2^* |  | *K*_F_ | *d* | *R^2^* |
| Highly charged LDHs  (Dilute) | 35.87 | 0.35 | 0.957 |  | 1.271 | 27.977 | 0.579 | 0.962 |  | 8.681 | 1.570 | 0.928 |
| Weakly charged LDHs  (Dilute) | 88.38 | 3.27 | 0.990 |  | 0.725 | 107.335 | 1.724 | 0.999 |  | 57.230 | 2.807 | 0.973 |
| Highly charged LDHs  (Concentrated) | 270.47 | 0.028 | 0.992 |  | - | - | - | - |  | - | - | - |
| Weakly charged LDHs  (Concentrated) | 141.07 | 0.23 | 0.998 |  | - | - | - | - |  | - | - | - |

**References:**

1. Giles, C. H., MacEwan, T. H., Nakhwa, S. N. & Smith, D. Studies in adsorption. Part XI. A System of classification of solution adsorption isotherms, and its use in diagnosis of adsorption mechanisms and in measurement of specific surface areas of solids. *J. Chem. Soc.* **846**, 3973−3993 (1960).
2. Rouquerol, F., Rouquerol, J., Sing, K. S. W., Llewellyn, P. & Maurin, G. *Adsorption by Powders and Porous Solids: Principles, Methodology and Applications* (Academic Press, Oxford, 1998).
3. Xu, Z. P. & Zeng, H. C. Abrupt structural transformation in hydrotalcite-like compounds Mg_1−x_Al_x_(OH)_2_(NO_3_)_x_·nH_2_O as a continuous function of nitrate anion. *J. Phys. Chem. B* **105**, 1743–1749 (2001).
4. Serna, C. J., Rendon, J. L. & Iglesias, J. E. Crystal-chemical study of layered [Al_2_Li(OH)_6_]^+^X^−^ nH_2_O. *Clays Clay Miner.* **30**, 180–184 (1982).
5. Kloprogge, J. T., Wharton, D., Hickey, L. & Frost, R. L. FT-Raman and FT-IR spectroscopic study of synthetic Mg/Zn/Al-hydrotalcites. *J. Raman Spectrosc.* **35**, 967–974 (2004).
6. Kloprogge, J. T., Hickey, L. & Frost, R. L., Infrared and Raman study of interlayer anions CO_3_^2−^, NO_3_^−^, SO_4_^2−^ and ClO_4_^−^ in Mg/Al hydrotalcite. *Am. Mineral.* **87**, 623–629 (2002).
